# Bead-based RNA Detection with Cas13a: Multi-scale Design and Validation

**DOI:** 10.64898/2026.07.27.739218

**Published:** 2026-07-28

**Authors:** Abdul M. Bhuiya, Siddhansh Agarwal, Carlos F. Ng, Melanie Ott, Sungmin Son, Daniel A. Fletcher

## Abstract

CRISPR-based assays offer sensitive and specific nucleic acid detection for a broad range of diagnostic and surveillance applications. While standard solution-based assays are simple, they typically lack the ability to detect multiple targets individually without the need for more complex instrumentation and multi-step workflows. Surface-based strategies offer greater potential for multiplexing since the identity of a target can be associated with a location on a slide or a specific bead, as in the case of DNA arrays or Luminex bead assays. However, the design principles for optimizing surface-based CRISPR assays have yet to be developed. Here we present a multi-scale analytical framework integrating nanoscale enzyme kinetics, microscale diffusion constraints, and macroscale assay parameters that we use to develop a bead-based Cas13a assay where the bead becomes fluorescent when the target RNA for that bead is detected. The assay, which we call SurfCas, uses surface-bound guide RNAs and reporter RNAs to detect one or more soluble target RNAs in a one-pot assay. We show that SurfCas has a sensitivity approaching bulk reactions, and we demonstrate multiplexed detection of target RNAs in a complex biological matrix. The design principles we establish for surface-based CRISPR assays offer guidance on how to further improve SurfCas sensitivity and scale multiplexed detection of target RNAs in a single reaction.

## Full Text Availability

The license terms selected by the author(s) for this preprint version do not permit archiving in PMC. The full text is available from the preprint server.

